# Crosstalk between colorectal cancer cells and cancer-associated fibroblasts in the tumor microenvironment mediated by exosomal noncoding RNAs

**DOI:** 10.3389/fimmu.2023.1161628

**Published:** 2023-05-10

**Authors:** Shichen Sun, Yanyu Zhang, Yubing Li, Linlin Wei

**Affiliations:** Department of Radiotherapy, Liaoning Cancer Hospital & Institute, Cancer Hospital of China Medical University, Shenyang, Liaoning, China

**Keywords:** colorectal cancer (CRC), exosomal noncoding RNAs (ncRNAs), cancer-associated fibroblasts (CAFs), tumor microenvironment (TME), exosomes

## Abstract

Colorectal cancer (CRC) is a common malignant tumor of the digestive system, and its morbidity rates are increasing worldwide. Cancer-associated fibroblasts (CAFs), as part of the tumor microenvironment (TME), are not only closely linked to normal fibroblasts, but also can secrete a variety of substances (including exosomes) to participate in the regulation of the TME. Exosomes can play a key role in intercellular communication by delivering intracellular signaling substances (e.g., proteins, nucleic acids, non-coding RNAs), and an increasing number of studies have shown that non-coding RNAs of exosomal origin from CAFs are not only closely associated with the formation of the CRC microenvironment, but also increase the ability of CRC to grow in metastasis, mediate tumor immunosuppression, and are involved in the mechanism of drug resistance in CRC patients receiving. It is also involved in the mechanism of drug resistance after radiotherapy in CRC patients. In this paper, we review the current status and progress of research on CAFs-derived exosomal non-coding RNAs in CRC.

## 1 Introduction

Colorectal cancer (CRC), a malignant tumor of the gastrointestinal tract, which derives from the epithelial cells of the colon or rectum, with the highest incidence and tumor-related mortality (1). The occurrence and progression of CRC are extremely connected with the patient’s age, gender, lifestyle, dietary habits, and genetic factors (2, 3). CRC is insidious and there are often no specific symptoms and signs in the early stages, A large proportion of patients are already at an advanced stage by the time they are diagnosed, and even have multiple metastases throughout the body, losing the opportunity for surgical treatment (4, 5). Moreover, about 30-40% of surgically resected CRC patients will develop recurrence and metastasis within 5 years (4). Despite the recent advances in treatment methods such as surgical treatment, chemotherapeutic agents, vascular targeting therapy, translational therapy, and local therapy (5–7) the overall 5-year OS of CRC patients is about 60%, and the overall prognosis is still poor (8, 9). As a result, elucidating the mechanisms of CRC progression and metastasis is virtually significant. Wherewith new strategies, the efficacy of systemic therapy can be improved.

Exosomes are small membranous vesicles with a diameter of 50-150 nm and contain a variety of biologically active molecules, such as proteins, nucleic acids, and lipids (10–14). Scientists first detected exosomes in mammalian mature erythrocytes secreted by intracellular multivesicular bodies fused to the cell membrane (15, 16). With further research, exosomes were discovered to be involved in cell-cell information transfer (17). Additionally, exosomes can regulate the biological functions of recipient cells by transmitting the genetic information they carry to the recipient cells (18). Associated with local or distant cells, tumor cells interact with the local or distant microenvironment, contributing to secondary malignant growth. Alterations in the tumor microenvironment play a vital role in the progression of malignant tumors, among which imbalance in the composition of immune cells, changes in the phenotype of fibroblasts and alterations in endothelial cells are characteristic of malignant lesions (19–21). Recently, there was an upward trend in the amount of studies, which have shown that never can people ignore the significance of exosomes to tumorigenesis, progression, associated immune responses, chemotherapy resistance and metastasis (22, 23). And exosome-derived non-coding RNAs also play an important role in CRC. Lnc-PCAT1 from CRC exosomes can promote epithelial to mesenchymal transition (EMT) and liver metastasis in CRC by regulating the activity of the Netrin-1-CD146 complex in circulating tumor cells (CTCs) and thus provide a new molecular target for the treatment of liver metastasis in CRC (24). Circ_00016174 in Doxorubicin (DOX)-resistant CRC tissues and cells Levels are upregulated in circ_0006174-enriched exosomes that enhance DOX chemoresistance in CRC by regulating the miR-1205/cyclin D2 (CCND2) axis (25). The CRC cell-derived exosome KCNQ1OT1 regulates the miR-30a-5p/biquitin-specific peptidase 22 (USP22) signaling axis and thus programmed death 1 (PD-1) ubiquitination and promotes immune escape from CRC (26).

**Figure 1:**
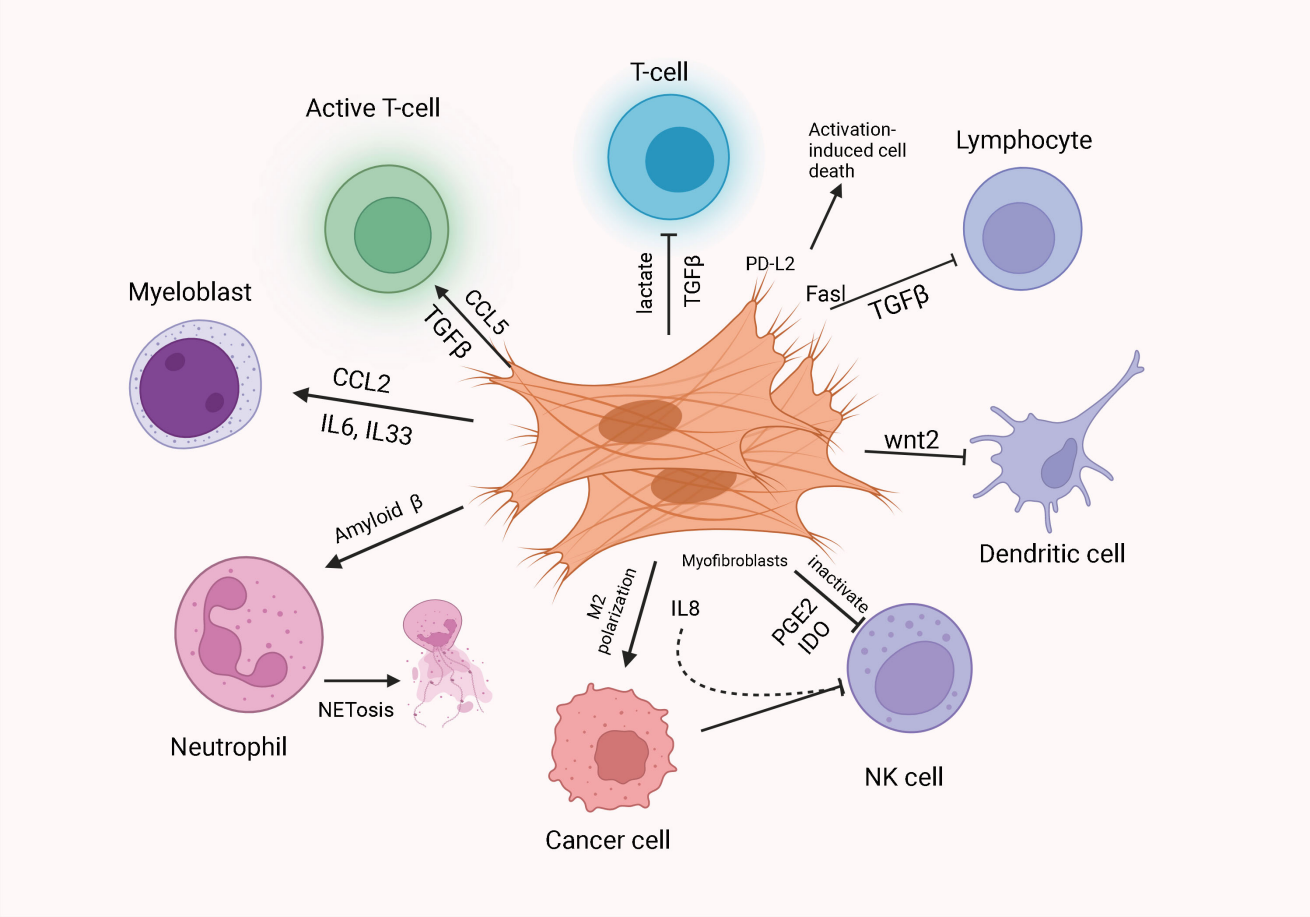
CAFs modulate the immunosuppressive microenvironment. CAFs can mediate the immunosuppressive microenvironment by promoting immunosuppression.CAFs can induce differentiation of neoplastic T cells into Tregs and recruit Tregs.CAFs can recruit MDSCs and enhance their immunosuppressive function.CAFs can promote NETosis and M2 polarization of TMA in TME. In addition, CAFs inhibit Th cell function and reduce CTL infiltration by secreting TGF-β. CAFs inhibit DC-mediated anti-tumor T cell responses and disable NK cell-mediated tumor killing through the secretion of PGE2 and IDO.

As crucial elements constituting the tumor microenvironment, cancer-associated fibroblasts (CAFs) are not only bound up with normal fibroblasts, but also can secrete a variety of substances involved in the regulation of the tumor microenvironment (TME) (27). There are several ways that CAFs have effects on tumor development. CAFs alter the extracellular matrix by synthesizing and degrading components and reshape its structure by cross-linking enzymes and proteases to establish a safety obstacle to tumors. CAFs also directly boost tumor cell proliferation and survival by secreting soluble mediators and promote angiogenesis (28, 29). CAFs also exert negative immunomodulatory impacts and allow tumors to evade immune surveillance (30). Recent studies illustrate that CAFs can secrete exosomes to enhance the metastatic and invasive ability of cancer cells by creating a microenvironment suitable for tumor growth (31, 32). In addition, the exosomes secreted by CAFs are known to be more potent than those secreted by tumors, mediating tumor immunosuppression, thus contributing to tumor development to a certain extent (33, 34) (**Figure 1**). In this paper, we discussed the origin of CAFs-related exosomes and reviewed the current status and progress of investigation into CAFs-derived related exosomal non-coding RNAs in CRC.

## 2 Overview of CAFs

**Figure 2:**
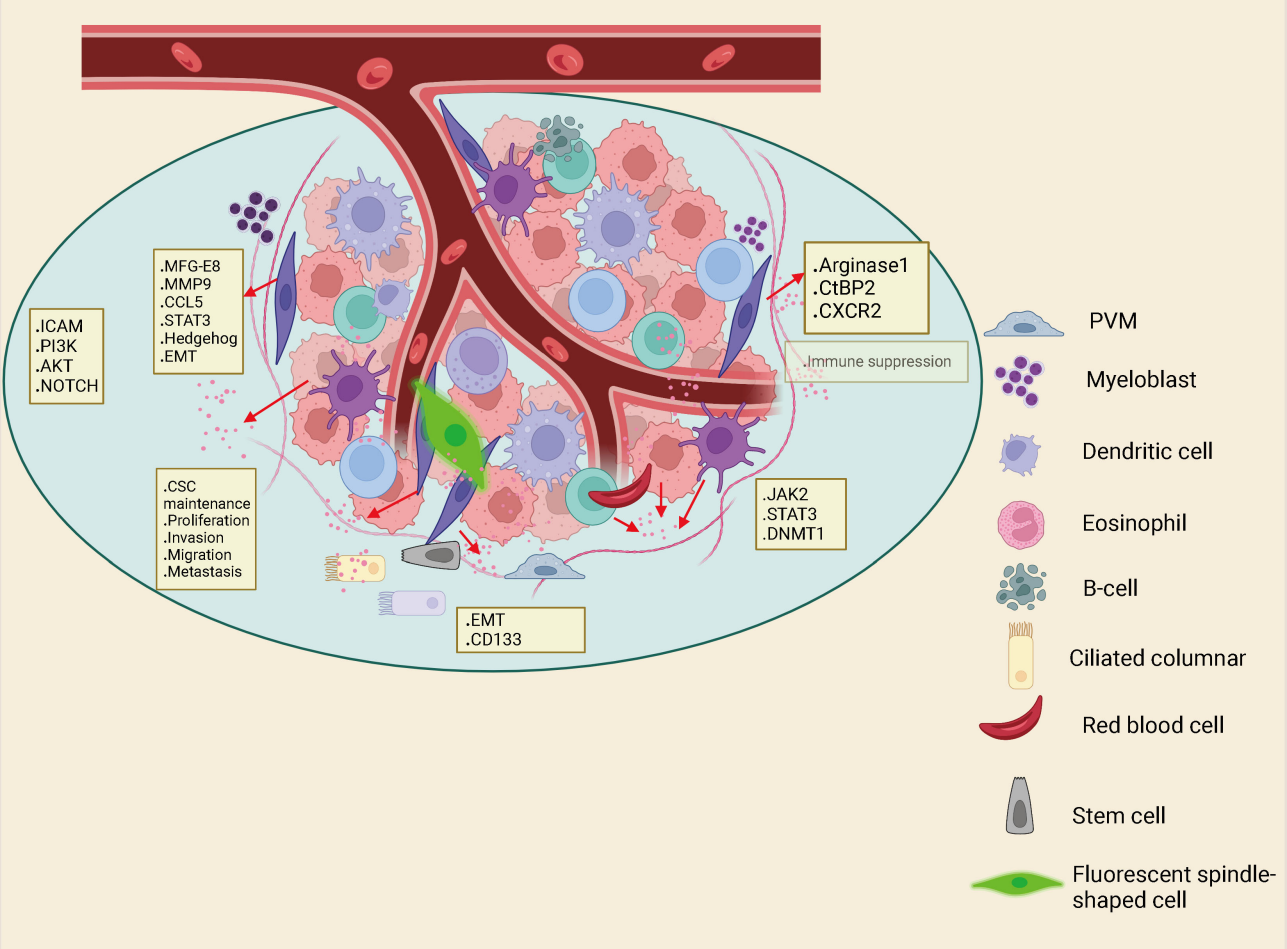
Interactions between immune cells, inflammatory cells, endothelial cells, adipocytes and fibroblasts in the Tumor microenvironment (TME) can also drive cancer stem cell maintenance.

TME is composed of tumor cells together with various mesenchymal cells and extracellular matrix (ECM). TME is a complex landscape, which is not only closely related to the growth and development of CRC, but also affects the treatment and prognosis of cancer patients. The components of the TME in CRC include tumor cells, blood vessels, the extracellular matrix, fibroblasts, lymphocytes, bone marrow-derived suppressor cells and signaling molecules. A series of cytokines, chemokines, growth factors, exosomes and other signal molecules interact to form a network in TME, enabling tumor to withstand and survive the increased pressure, leading to cancer metastasis, immunosuppression, abnormal angiogenesis and drug resistance (35). Tumor mesenchymal cells are comprised of fibroblasts, vascular endothelial cells, inflammatory/immune cells, mesenchymal stem cells, adipocytes (36, 37) (**Figure 2**). CAFs are fibroblasts that are activated in the TME. Among all mesenchymal cells that constitute TME, CAFs are the most abundant tumor mesenchymal cells. Despite the widespread presence of CAFs in the tumor mesenchyme, their role in tumor development has been under-recognized. In recent years, abundant evidence has indicated that CAFs produce tumor-supporting ECM, facilitate the growth, expansion and spread of pre-tumor epithelial cells, create a comfortable environment for emerging malignant cells, and are vital drivers of tumor progression in many organs (38, 39). In TME, CAF can regulate the biological behavior of tumor cells and other mesenchymal cells through cell-to-cell contacts; and can effect tumorigenesis and progression *via* release of large amounts of regulatory factors to synthesize and remodel the ECM (40, 41). In CRC, previous studies have showed that CAFs are the main cellular constituents of stroma associated with primary and metastatic CRC (42, 43). Compared with normal mucosa, the number of myofibroblasts in CRC was significantly increased. Studies have shown that fibroblasts in the lamina propria of colon polyps α- SMA − becomes α- SMA^+^, which indicates that interstitial fibroblasts of lamina propria show myofibroblast differentiation (44). CAF can also undergo reprogramming of lipid metabolism and secrete lipid metabolites, which can be absorbed by CRC cells and promote migration. This is partly caused by the overexpression of vimentin and the down-regulation of E-cadherin (45). Although CRC contains a subset of high-level stromal cells, the CAF in CRC is still relatively insufficient. Therefore, more understanding on the interaction between CAF and tumor cells to summarize the current knowledge about the role of CAF in cancer.

### 2.1 Sources of CAFs

The tumor microenvironment consisting of mesenchymal cells and extracellular matrix plays a significant role in the development of tumor formation (46). The distinctive features of the mesenchymal microenvironment of tumor cells are altered ECM components, increased microvascular density and inflammatory cell numbers, and the presence of activated fibroblasts. These activated fibroblasts are called myofibroblasts or CAFs. Studies have shown that CAFs have several main sources (47). They are formed by resident fibroblasts induced to differentiate in response to various cytokines secreted by cancer cells, such as platelet-derived factor, transforming growth factor β (TGF-β) (48, 49). Myofibroblasts have obvious similarities to vascular smooth muscle cells and perivascular cells, which in turn may be formed from vascular beds (50). Recent researches have shown that epithelial tumor cells themselves are capable of transforming into mesenchymal cells *via* the EMT pathway (51). Some of the bone marrow mesenchymal stem cells (MSCs) have been shown to be able to transform into mesenchymal cells through the EMT pathway. Some bone marrow MSCs can also differentiate to form CAFs (52). In conclusion, most CAFs originate from peripheral mesenchymal fibroblasts, a small fraction from vascular smooth muscle cells, and a much smaller fraction from perivascular cells (53).

### 2.2 Heterogeneity of CAFs

Studies have shown that cell phenotypic differences are the main manifestation of the heterogeneity of CAFs, and the phenotypic transition of CAFs has a temporal as well as a spatial character which refers to the different phenotypes of fibroblasts in different parts of the tissue and the differentiated phenotypes of the same fibroblast in various parts of the tissue (50, 51). With the development of related technologies, it has become possible to quantitatively analyze cellular transcriptome differences at the single cell level. It has been shown that CAFs can be classified into mCAF subpopulation, dCAF subpopulation, vCAF subpopulation, and cCAF subpopulation based on the genes of CAFs subpopulations (54). The mCAF subpopulation is converted from resident tissue fibroblasts, the dCAF subpopulation is derived from tumor epithelial mesenchyme, the vCAF subpopulation is converted from perivascular cells, and the cCAF subpopulation overlaps with vCAF, while having a strong proliferative capacity.

### 2.3 Interaction between CAFs and tumor cells

**Figure 3:**
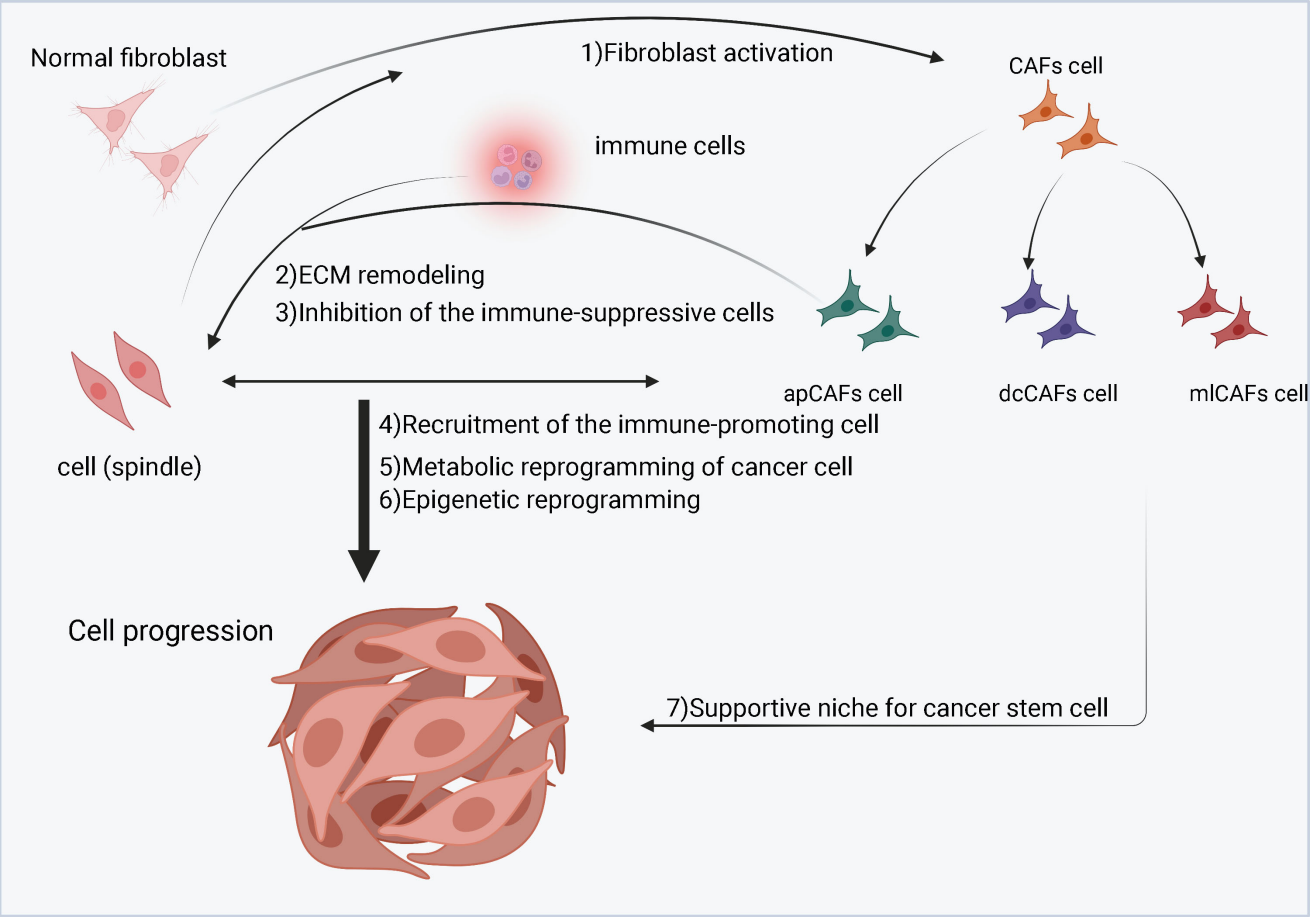
A schematic diagram of cancer-associated fibroblasts (CAFs) in the tumor microenvironment (TME). Cancer cells stimulate normal fibroblasts to become CAFs. CAFs are divided into three subgroups, namely inflammatory CAFs (iCAFs), myofibroblastic CAFs (myCAFs) and antigen-presenting CAFs (apCAFs). CAFs, especially myCAFs, promote the remodeling of the extracellular matrix (ECM). CAFs (iCAFs and myCAFs) and cancer cells interact leading to metabolic reprogramming of cancer cells. In contrast, proliferation of apCAFs leads to the recruitment of immunosuppressive cells and inhibits the growth of immune promoting cells.

Studies have shown that CAFs can interact with neighboring tumor cells (**Figure 3**). Normal fibroblasts can inhibit tumor cell growth and promote differentiation of tumor cells to their malignant phenotype (47, 55, 56). In contrast, CAFs can significantly stimulate tumor cell growth when co-cultured with tumor cells (57). CAFs can make tumor cells easily invade blood vessels by establishing and remodeling the extracellular matrix structure (58–60). They can promote tumor progression by secreting a host of growth factors, cytokines and chemokines that interact with tumor cells or other stromal cells (61–63). What is more, CAFs are considered as salient targets for the development of novel anticancer drugs (64, 65). The action of tumor cells on CAFs is mainly achieved through cytokines. factors such as TGF-β, PDGF, IGF and colony stimulating factor (CSF) can induce mesenchymal responses (66, 67). Among them, TGF-β, platelet-derived growth factors (PDGF), insulin-like growth factor (IGF) and extracellular matrix metalloproteinase inducer (EMMPRIN) are considered to be key factors in the process of tumor formation.

## 3 Overview of exosomes

Exosomes are a class of lipid bilayer vesicles 30-150 nm in diameter that encapsulate a variety of proteins, lipids, nucleic acids, and other metabolites that can be secreted by most types of cells and are widely present in body fluids such as urine, emulsions, tears, and cerebrospinal fluid (68, 69). Exosomes can be involved in numerous physiological activities, including intercellular communication, mammalian reproduction, and immune regulation, which play an important role in the pathological progression of diseases such as cardiovascular disease, neurodegeneration, and cancer (70, 71). In recent years, the role of exosomes in communication between tumor cells and fibroblasts has become a hot research topic. Meanwhile, more and more studies have used exogenous RNA and protein as new biomarkers of CRC. Proteome analysis identified many proteins that were differentially expressed in CRC cell exosomes. A study found that long non-coding RNAs (lncRNAs) are differentially distributed in the exosomes from normal cells and CRC-associated fibroblasts (72). Similarly, another study also found that different lncRNAs were classified differently as secretions secreted by CRC cells (73).

### 3.1 Exosome biosynthesis

**Figure 4:**
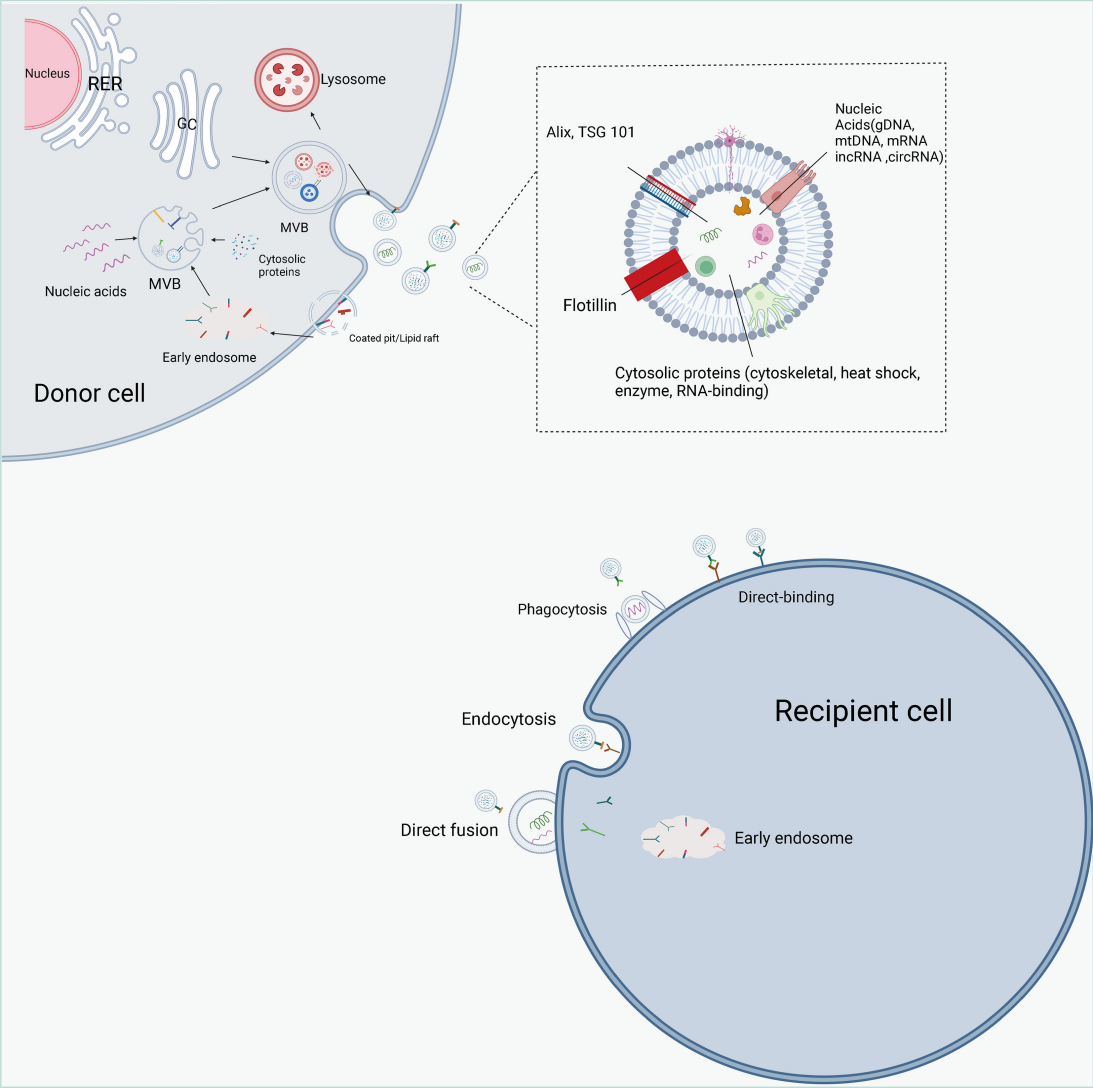
The biogenesis, contents, and internalization of exosomes. Exosome formation includes initiation, endocytosis, multivesicular body (MVBs) formation and secretion. intracellular trafficking of MVBs is mediated by Rab GTPase. The fusion of multivesicular bodies with the plasma membrane is facilitated by SNAREs. There are three types of interactions between exosomes and cells: direct binding of membrane proteins on exosomes and target cells, which then triggers an intracellular signaling cascade; transport of exosomes to target cells by fusing with cell membranes to deliver their contents; and phagocytosis of exosomes by cells and degradation by lysosomes to release signaling molecules. Exosomes are rich in proteins, lipids, and non-coding RNAs, and have a variety of specific proteins on their surface, such as Alix, TSG101.

Classical exosome formation begins with the formation of secretory endosomes by invagination of the cytoplasmic membrane, which subsequently form intraluminal vesicles (ILVs) in an outgrowth fashion and contain various cytoactive substances within the vesicles (74, 75) (**Figure 4**). Secretory endosomes containing multiple ILVs are called multivesicular bodies (MVBs), and with acidification, multivesicular bodies (MVBs) maturate and fuse with the plasma membrane, transferring ILVs outside the cell and eventually forming exosomes (76, 77). The Endosomal sorting complex required for transports (ESCRT) is involved in classical exosome formation. The ESCRT complex can be broadly divided into four components of ESCRT0, ESCRTTI, ESCRTII and ECRTIII (78–80). During the biogenesis of MVBs, the cellular material to be transported is ubiquitinated and the ESCRT0 complex is recruited to the membrane of the secretory endosome. Immediately thereafter, ESCRTI and ESCRTII allow the secretory endosomes to form a budding pattern and encapsulate cellular active substances such as proteins and nucleic acids; the vesicles detach from the cell membrane in the presence of the ECRTIII complex (81, 82). Moreover, other proteins contribute to exosome biogenesis, including apoptosis-linked gene-2 interacting protein X (Alix), vacuolar protein sorting 4 (Vps4), tumor susceptibility gene 101 (TSG101) and chromatin modifying protein 4 (CHMP4) (83, 84). Currently, the common biomarkers of exosomes are the four membrane penetrating proteins (CD82, CD81, CD63 and CD9), heat shock proteins (HSP70, HSP90) and related proteins involved in membrane transport and fusion (membrane linker and Rab) (85).

### 3.2 Exosome biological functions

Exosomes were once thought to be the only way for cells to excrete waste products, but subsequent researches have demonstrated that exosomes function as transporters of substances, transmitters of information and biomarkers in the physiological and pathological processes of the organism (86–88). Exosomes are extremely significant to tumor growth, metastasis, angiogenesis and immune regulation, metabolism, and drug resistance by delivering a variety of biomolecules that mediate signal transduction (89, 90). Exosomes, as effective biomarkers of diseases, especially cancer, have become a new research field. It is reported that the exosome miRNA-103, the tripartite motif containing three proteins, the glypican-1 protein and the tyrosine kinase substrate protein regulated by hepatocyte growth factor may be used to detect liver cancer, gastric cancer, pancreatic cancer and colon cancer (91). In addition, Some exosomes are involved in the regulation of CRC metastasis, drug resistance and relapse. These exocrine molecules can affect the prognosis of CRC patients and may be useful biomarkers for these individuals (92).

## 4 CRC cell-derived exosomal ncRNAs activate CAFs

A variety of molecules have been identified in exosomes, including proteins, lipids and nucleic acids (93). The bilayer membrane of exosomes protects these molecules from protease, nuclease and other environmental influences (94). In addition, these molecules in exosomes are selectively packaged, secreted and transferred between cells, and highly variable, depending on parental cells and pathophysiological conditions (95). More and more evidence shows that the exocrine body is rich in ncRNA, including microRNA (miRNA), long non-coding RNA (lncRNA), circular RNA (circRNA), piwi interaction RNA (piRNA) and small non-coding RNA derived from tRNA, which play an important role in various pathophysiological processes, especially in cancer (96–98). In recent years, the development of next-generation sequencing technology has led to the proliferation of newly discovered non-coding RNA (ncRNA), such as microRNA (miRNA), linear long non-coding RNA and circular non-coding RNA. Unlike messenger RNAs (mRNAs), ncRNAs do not encode proteins, but act as epigenetic regulators (99), post-transcriptional modifiers (100) and translation coordinators of gene expression (101). With the discovery and further research of ncRNA in exosomes, many new functions and applications have emerged, from new ways of intercellular communication to promising biomarkers of diseases, and considering the biocompatibility of exosomes, there may be new therapeutic applications.

Studies have shown that exosomal ncRNAs of CRC cell origin activate CAFs (**Table 1**). LINC01915 expression is decreased in CRC tissues as well as in CAFs, and low expression of LINC01915 predicts a poor prognosis for CRC patients. Overexpression of LINC01915 in CRC cells inhibits tumor angiogenesis, CAF activation, and normal fibroblasts (NFs) uptake of tumor-derived extracellular vesicles (EVs). Mechanistic experiments showed that LINC01915 could regulate the miR-92a-3p/KLF4/CH25H axis and thus prevent angiogenesis and the conversion of NFs to CAFs and significantly inhibit the malignant progression of CRC (102) to inhibit the uptake of NFs to CRC-derived EVs. Expression of miR-1246 was significantly elevated in CAF-like fibroblasts compared to normal fibroblasts, and miR-1246 secreted by CRC cells could be utilized by neighboring fibroblasts and used for CAF reprogramming. In addition, CAF-like fibroblasts can also secrete miR-1246 into CRC cells and promote cell migration by activating Wnt/β-catenin signaling, and high miR-1246 expression also predicts poor prognosis in CRC patients (103). Investigations have indicated that epithelial CRC inhibits myofibroblast differentiation by secreting EVs and transferring miR-200 (miR-200a/b/c-141) into recipient fibroblasts and by targeting zinc-finger E-box-binding (ZEB) (104). C-X-C motif chemokine receptor 7 (CXCR7) treatment of CRC cells increased the expression levels of miR-146a-5p and miR-155-5p in their exosomes. In addition, CRC cell-derived exosomal miR-146a-5p and exosomal miR-155-5p were delivered to CAFs and promoted the activation of CAFs by targeting cytokine signaling 1 (SOCS1) and zinc finger and BTB domain containing 2 (ZBTB2)-containing cells and regulating JAK2-STAT3/NF-κB signaling. Activated CAFs can in turn encourage the invasive capacity of CRC cells (105).

**Table 1:** Potential effects of exosomal ncRNAs derived from CRC cells on CAFs.

NcRNAs	Expression	Biological function	Targets	Reference
LINC01915	Down	Inhibition of tumor angiogenesis and activation of CAF	miR-92a-3p/KLF4/CH25H	(102)
miR-1246	Up	Promote CAF reprogramming	Wnt/β-catenin	(103)
miR-200 family	Up	Inhibit the differentiation of myofibroblasts	ZEB	(104)
miR-146a-5p		Promote the activation of CAFs	SOCS1/ZBTB2-JAK2–STAT3/NF-κB	(105)
miR-155-5p				

## 5 Effect of CAFs-derived exosomal ncRNAs on CRC cells

### 5.1 CAFs-derived exosomal ncRNAs modulate the effect of chemotherapy in CRC

5-Fluorouracil, oxaliplatin and methotrexate are commonly utilized chemotherapeutic agents as the treatment of CRC, but a majority of patients develop intrinsic and acquired resistance (106, 107). exosomal ncRNAs of CAFs origin modulate the efficacy of chemotherapy in CRC (**Table 2**). h19 expression is significantly higher in tumor tissues of mice with colitis-associated cancer (CAC), and high expression of h19 is also positively associated with CRC The high expression of H19 was also positively correlated with lymph node metastasis in CRC. Overexpression of H19 significantly promoted the stemness of CSC cells. To explain it further, H19 was also enriched in CAFs-derived exosomes and could be phagocytosed by CSC and CRC cells, thus promoting the stemness of CSC and drug resistance of CRC cells. Mechanistic results showed that H19 could activate the β-catenin pathway by binding miR-141. In a nutshell, CAFs can boost tumor stemness and drug resistance by transferring exosomal H19 into CRC cells and activating the β-catenin pathway through binding miR-141 (108). It has been shown that lnc-CCAL is enriched in the exosomes of CAFs and can be delivered to CRC cells and inhibit apoptosis and promote chemoresistance by activating the β-catenin pathway. The results of mechanistic experiments demonstrated that CCAL could interact with HuR to increase the expression of β-catenin. All in all, exosomal lnc-CCAL derived from CAFs in the colorectal tumor mesenchyme can metastasize into CRC cells and promote resistance to oxaliplatin (Oxa) (109). MiR-24-3p expression was significantly increased in both colon cancer (CC) tissues and cells, and concomitant overexpression of miR-24-3p after treatment of CC cells using methotrexate (MTX) promoted cell viability and colony-forming ability and inhibited apoptosis. In addition, miR-24-3p was also enriched in CAFs-derived exosomes and could be transferred to colon cancer cells. Under MTX treatment, treatment of colon cancer cells with CAFs-derived exosomal miR-24-3p promoted tumor growth and malignant progression, and mechanistic experiments showed that miR-24-3p accelerated the resistance of colon cancer cells to MTX by targeting the caudal-related homeobox transcription factor 2 (CDX2)/HPEH regulatory axis (110). Researchers identify MiR-181d-5p, which is enriched in CAFs, as a miRNA associated with 5-Fluorouracil (5-FU) sensitivity. m6A modification and methyltransferase-like 3 (METTL3) expression are significantly elevated in CRC patient tissues, and METTL3 promotes the methylation modification of miR-181b 5p and its expression *via* DiGeorge syndrome critical region 8 (DGCR8). The results of mechanistic experiments showed that CAFs-derived exosomes could inhibit 5-FU sensitivity in CRC cells *via* METTL3/miR-181d-5p/neurocalcin delta (NCALD) axis (111) CricN4BP2L2 was enriched in the exosomes of CAFs and could be delivered to LoVo cells and promote oxaliplatin resistance and stemness in LoVo cells while inhibiting apoptosis. The outcome of mechanistic experiments showed that cricN4BP2L2 could regulate the PI3K/AKT/mTOR regulatory axis by binding to EIF4A3. In conclusion, CAFs-exo-cricN4BP2L2 allows regulating the EIF4A3/PI3K/AKT/mTOR pathway (112). To promote stemness and oxaliplatin resistance in CRC cells. The expression levels of miR-200b-3p were significantly lower in CRC tissues than in normal control tissues, and miR-200b-3p expression levels were also lower in hypoxic CAFs than in normoxic CAFs. Compared with the exosomes of normoxic CAFs, the exosomes of hypoxic CAFs could target HMGB3 and bcatenin/c-Myc regulatory axis by secreting miR-200b-3p and thus promote the therapeutic effect of 5-FU on CRC *in vivo* (113).

**Table 2:** Potential effects of exosomal ncRNAs derived from CAFs on chemotherapy of CRC.

NcRNAs	Expression	Biological function	Targets	Reference
H19	Up	Promote the stem cell property of CSC and the drug resistance of CRC cells	miR-141/β-catenin	(108)
Lnc-CCAL	Up	Inhibition of cell apoptosis and promotion of Oxa chemoresistance	HuR/β-catenin	(109)
MiR-24-3p	Up	Promote tumor growth, malignant progression and MTX resistance	CDX2/HPEH	(110)
MiR-181d-5p	Up	Inhibition of 5-FU sensitivity	METTL3/miR-181d-5p/NCALD	(111)
CricN4BP2L2	Up	Promote oxaliplatin resistance and stem cell property and inhibit cell apoptosis	EIF4A3/PI3K/AKT/mTOR	(112)
miR-200b-3p	Down	Promote sensitivity of 5-FU	HMGB3/bcatenin/c-Myc	(113)

### 5.2 CAFs-derived exosomal ncRNAs promote radiation therapy resistance in CRC

Studies have shown that CAFs-derived exosomal ncRNAs promote radiation therapy resistance in CRC (114, 115). MiR-590-3p expression was significantly increased in CRC tissues and cell lines and enriched in CAFs. Treatment of CRC cells with CAF-derived exosomal miR-590-3p increased cell survival and the p-PI3K/PI3K and p-AKT/AKT ratios and decreased the expression of cleaved PARP, cleaved protease 3 and gH2AX in the cells. Moreover, exosomal miR-590-3p significantly stimulated tumor growth in CRC mice after radiotherapy (116). It was confirmed that miR-31 expression was significantly higher in CAFs than in normal colorectal fibroblasts (NFs), and overexpression of miR-31 in CAFs inhibited the expression of autophagy-related genes Beclin-1, ATG, DRAM and LC3. Furthermore, miR-31 in CAFs can be delivered to CRC cells and promote cell proliferation, invasion, and radiosensitivity, and inhibit apoptosis (117) MiR-93-5p expression was significantly higher in CAFs-derived exosomes than in normal fibroblasts (NFs), and treatment of SW480 cells with exosomal miR-93-5p promoted cell proliferation and protected them from radiation-induced apoptosis. The outcome of mechanistic experiments showed that miR-93-5p could inhibit forkhead box protein A1 (FOXA1) binding to the promoter of transforming growth factor beta3 (TGF-β3) and promote the nuclear accumulation of TGFβ3 by targeting FOXA1 and suppressing its expression. Besides, exosomal-miR-93-5p derived from CAFs promotes tumor growth in irradiated nude mice (114).

### 5.3 CAFs-derived exosomal ncRNAs promote CRC metastasis and progression

Malignant growth and metastasis of CRC is a multistep, multistage, multigene regulatory process, and exosomal ncRNAs derived from CAFs can promote malignant progression of tumors by regulating proliferation, migration and invasion of CRC cells (**Table 3**). Cell proliferation, migration, and cell cycle can be promoted by the use of CAFs co-cultured with CRC cells (130). The results of mechanistic experiments illustrated that CAFs delivered UCA1 to CRC cells and promoted the upregulation of mTOR, while the UCA1/mTOR regulatory axis inhibited the expression of p27 and miR-143 and promoted the expression of Cyclin-D1 and Kirsten rat sarcoma (KRAS) thus promoting the malignant progression of CRC (118) miR-224-5p expression was significantly increased in CRC and targeted to suppress SLC4A4 expression. Furthermore, miR-224-5p was also enriched in CAFs-derived EVs, which could transfer miR-224-5p into CRC cells and promote cell proliferation, migration, invasion, and inhibition of apoptosis (119). Hypoxia can induce the secretion of circEIF3K in CAFs exosomes. Also, cell proliferation, invasion and tube formation can be facilitated through the use of CAFs exosomes co-cultured with CRC cells. Mechanistic findings demonstrate that hypoxia induces the secretion of circEIF3K from CAFs into CRC cells and promotes malignant growth and metastasis of CRC by regulating the miR-214/PD-L1 axis (115). It was shown that miR-135b-5p was enriched in the EVs of CAFs and could be sent to COAD cells to promote malignant cell behavior as well as COAD cell-mediated HUVEC proliferation, migration and angiogenesis. The consequences of mechanistic experiments showed that miR-135b-5p could target FOXO1 and thus promote malignant progression of COAD (120). Prior study has shown that treatment of CRC cells with exosomes derived from CAFs promotes cell proliferation, migration, tube-forming ability and inhibits apoptosis of CRC cells. The results of mechanistic experiments showed that CAFs could deliver circN4BP2L2 to CRC cells through secreted exosomes and inhibit CRC cell proliferation and migration by regulating the miR-664b-3p/HMGB3 pathway (121). There’s a significant increase in SNHG3 expression in CRC cells and CAFs-derived exosomes, while incubation of CRC cells using CAFs-EVs facilitated cell proliferation. Mechanistic experiments turned out that CAFs-EVs can carry SNHG3 into CRC cells and upregulate HuR expression by competitively binding to miR-34b-5p, which in turn promotes the binding of HuR and HOXC6 and enhances the transcription of HOXC6 and promotes the malignant progression of CRC (122). CircSLC7A6 expression recorded a significant growth in CRC tissues and promoted proliferation, invasion and inhibited apoptosis of CRC cells, while CAFs could promote malignant progression of tumors by secreting exosomal circSLC7A6 into CRC cells and regulating CXCR5 expression. Treatment of CRC cells with bitter ginseng alkaloids significantly inhibited cell proliferation and invasion and increased apoptosis by inhibiting the secretion of exosomal circSLC7A6 by CAFs (123). Compared to NFs-exo, the expression of miR-17-5p was significantly higher in CAFs-exo. In addition, CAFs can deliver miR-17-5p from parental CAFs to CRC cells *via* the exosomal pathway. The results of mechanistic experiments showed that miR-17-5p could regulate the runt-related transcription factor3 (RUNX3)/MYC/TGF-β1 signaling axis to enhance CRC metastasis. Sustained autocrine TGF-β1 activates CAF and releases more exosomal miR-17-5p into CRC cells, thus forming a positive feedback loop for CRC progression (124) LINC00659 expression was significantly increased in the exosome of CAFs, which could transfer exosomel LINC00656 to CRC cells and promote cell proliferation, migration, invasion and EMT progression. Mechanistic experiments showed that LINC00659 binds to miR-342-3p and promotes the expression of ANXA2 (Annexin A2) (125). In CRC tissues and cells, MiR-135b-5p expression was upregulated, whereas thioredoxin-interacting protein (TXNIP) expression was downregulated. caF-exo and caF-exos upregulated miR-135b-5p, promoted growth *in vivo*, proliferation, migration and invasion *in vitro*, inhibited CRC cell apoptosis, and promoted HUVEC angiogenesis. It turns out that txNIP is a miR-135b-5p target, and overexpression of TXNIP attenuated the pro-CRC effect of exosomal miR-135b-56. It has been reported that CAF exosomes promote CRC cell growth and angiogenesis by inhibiting TXNIP upregulation of miR-135b-54 (126). WEE2-AS1 expression is significantly increased in CAFs-derived exosomes, and high levels of WEE2-AS1 also predict poor prognosis in CRC patients. CAFs deliver exosomal WEE2-AS1 to CRC cells and promote cell proliferation as well as tumor formation and progression. The results of mechanistic experiments show that WEE2 antisense RNA 1 (WEE2-AS1) inhibits the Hippo pathway and thus CRC cell growth by binding MOB1A and promoting its degradation (127) CAFs-Exo can deliver miR-625-3p to CRC cells and promote CRC cell migration, invasion, EMT and chemoresistance by inhibiting the CELF2/WWOX pathway (128). MiR-181b-3p expression was enhanced in CRC, and exosomes using CAFs promoted miR-181b-3p expression after co-incubation with CRC cells. In addition, treatment of CRC cells with exosomes which are derived from CAFs significantly, could be a great boost to cell proliferation and migration and a decrease to the proportion of apoptotic cells. The results of mechanistic experiments showed that miR-181b-3p could promote the malignant progression of CRC by targeting and inhibiting the expression of SNX2 (129).

**Table 3:** Potential effects of exosomal ncRNAs derived from CAFs on malignant progression of CRC.

NcRNAs	Expression	Biological function	Targets	Reference
UCA1	Up	Promote cell proliferation, migration and cell cycle	mTOR/p27/miR-143/Cyclin-D1/KRAS	(118)
miR-224-5p	Up	Promote cell proliferation, migration, invasion and inhibit cell apoptosis	SLC4A4	(119)
circEIF3K	Up	Promote cell proliferation, invasion and tube formation	miR-214/PD-L1	(115)
miR-135b-5p	Up	Promote the malignant behavior of CRC cells	FOXO1	(120)
circN4BP2L2	Up	Promote cell proliferation, migration and tubular formation and inhibit the apoptosis of CRC cells	miR-664b-3p/HMGB3	(121)
SNHG3	Up	Promote the malignant progression of CRC	miR-34b-5p/HuR/HOXC6	(122)
CircSLC7A6	Up	Promote the proliferation and invasion of CRC cells and inhibit cell apoptosis	CXCR5	(123)
miR-17-5p	Up	Promote the metastasis of CRC	RUNX3/MYC/TGF-β1	(124)
LINC00659	Up	Promote cell proliferation, migration, invasion and EMT progress	miR-342-3p/ANXA2	(125)
miR-135b-5p	Up	Promote CRC cell growth and angiogenesis	TXNIP	(126)
WEE2-AS1	Up	Promote cell proliferation and tumor formation and progression	MOB1A/Hippo	(127)
miR-625-3p	Up	Promote the migration, invasion, EMT and chemoresistance of CRC cells	CELF2/WWOX	(128)
miR-181b-3	Up	Enhance cell proliferation and migration and reduce cell apoptosis	SNX2	(129)

### 5.4 The current application of CAFs-related exosomal ncRNAs in the diagnosis and treatment of CRC

Exosomes function as messengers in the communication between CRCs and CAFs, whose transported cargoes are relevant to their parental cells and can be used as markers to determine disease progression (131). It has been reported that plasma exosomes extracted from CRC patients and found by assay that the expression of miR-590-3p was significantly higher in plasma from CRC patients compared to healthy control plasma, and the expression of exosomal miR-590-3p was reduced after tumor resection. In addition, exosomal miR-590-3p expression levels were significantly higher in radioresistant CRC patients than in radiosensitive patients (116). Previous study also showed the results of isolated and examined the expression levels of exosomal WEE2-AS1 in plasma samples from 50 CRC patients and 50 healthy subjects. Analysis of clinical characteristics of CRC patients revealed that exosomal WEE2-AS1 expression correlated with CEA, tumor size and TNM stage, and high expression of exosomal WEE2-AS1 also predicted poor overall survival and disease-free survival in CRC patients (127).

## 6 Conclusions and prospects

CRC, whose trend of incidence and mortality worldwide is increasing, is a common malignant tumor of the gastrointestinal system. The development of colorectal cancer is often a complex process involving multiple factors, stages, and links, in which CRC cells interact and evolve synergistically with multiple components of TME, thus promoting its development (132, 133). The extensive, multi-level interactions between tumor cells and mesenchymal cells provide TME to support tumor survival, growth, and metastasis (134–136). CAFs, one of the most copious mesenchymal cells in TME, are in an activated state and are phenotypically and functionally altered to interact with immune cells and cancer cells through multiple signaling pathways, including autocrine and paracrine, to form complex molecular networks and perform their biological functions (137, 138).

Despite the current research on CAFs has been fruitful, many questions remain ambiguous. For example, are the original sources of CAFs different in different types of cancer? Which subtypes of CAFs exist in TME? Do these subtypes of CAFs with different phenotypes and immune functions have different cellular origins? What are their specific markers? Why do different subpopulations of CAFs have opposite results on tumor regulation. Currently, large-scale randomized clinical trials remain a major gap in the field of targeted CAFs therapeutics. Consequently, a large number of original studies targeting CAFs are needed to further elucidate their clinical value and impact on cancer progression (130, 139). Exosomes are a communication tool between multiple cells, and CAFs and tumor-derived exosomes are more extensively studied. Nevertheless, the mechanism of action of CAFs-derived exosomes in malignancies still lacks in-depth studies (126, 140), especially in CRC, where relevant studies are relatively vacant. As a consequence, research on exosomes of CAFs origin has a broad prospect, and an in-depth study of the relationship between the CRC microenvironment, CAFs and exosomes of related origin will be more beneficial to understand the malignant process, drug resistance and other mechanisms of action in CRC for the benefit of more patients.

To sum up, exosomes are released from CRC cells and CAFs, playing a crucial role in regulating cancer progression in the primary tumor microenvironment. Exosomes of CAFs origin can regulate the processes of chemoresistance, radiation resistance, and malignant progression in CRC, while exosomes of CRC cell origin can better regulate fibroblasts to better assist tumor cells. Exosomes secreted by CRC cells and CAFs cells (ncRNA encapsulated in exosomes) can be used as biomarkers for auxiliary diagnosis of CRC metastasis, drug resistance and prognosis, and have potential clinical applications.

## Author contributions

LW: Conceptualization, methodology, software data curation, writing-original draft preparation. YL, YZ and SS: Visualization, investigation, supervision. writing-reviewing and editing. All authors contributed to the article and approved the submitted version.
